# Salmagundi

**Published:** 1888-06

**Authors:** 


### SALMAGUNDI.

EDITED BY E. G. BETTY, D.D.S.

DR. G. STICKER is of the opinion that contrary to the prevalent belief, saliva is an important element in gastric digestion by promoting the secretion of the gastric juice.

AN exchange says : "The painful headache frequently preceeding or accompanying menstruation, is often relieved by a teaspoonfull of powdered guarana and ten grains of bicarbonate of soda, in one dose, taken in a glassfull of water or milk." This is a hint that the dentist can often use to his own advantage and that of the patient. Uterine troubles are more frequently the cause of nervousness and hysteria than we imagine and a judicious question or two will often save pain to the patient and time for both if sittings are arranged so as not to conflict with the period of the menopause.

THE Dental Section of the Thirty-Ninth Annual Meeting of the American Medical Association was well attended and accomplished some good work of a scientific character. Three papers especially are deserving of careful study, *viz:* Dr. Talbott's on "Etiology of Irregularities of the Teeth," "Development of the Superior Maxilla in the Idiot, Deaf and Dumb, and Blind, and Insane;" Dr. Marshall's on "Fracture of the Superior Maxilla and Upper Bones of the Face, Treatment by Aid of the Dental Splint with two Illustrations," and "Dentogeny" by Dr. Brittan, together with the written discussion by Dr. Fletcher. These papers reflect great credit upon their authors and upon the section before which they were read. It is greatly to be regretted that the validity of the delegate certificates from the Mississippi Valley and Cincinnati Dental Societies should have been questioned in print by a contemporary, the effect being to deter the delegate as well as others who would have registered and added their mite to the section. Personal spite, pique and enmity should never animate antagonism to "the good of the order," the effect is depressing upon members of the profession and baneful in its influence upon the estimation in which the profession is held by that of medicine and the laity.

The Section on "Oral and Dental Surgery" is destined to do more good than two International Dental Congresses, for the reason that it is the medium through which we shall gain the ear of the medical profession at large throughout this great country, and by that means an increased demand for the services of the dentist will spring up when the public in general is advised by the physician to consult him. These small differences of opinion, which for a time seem weighty, are begotten in ignorance, narrow-mindedness, and jealousy; like the mushroon, they have their hour betwixt two days, but when the bright sun of science and truth sheds forth its rays, they dry up, fade away, and they and their progenitors sink out of sight never to be thought of again.

---

"THEY are both ladies prominent in the most refined, religious and social circles to be found in Kentucky and reside not a hundred miles from Stanford. One, whom we will call Mrs. A. was a modest matron, and desirous of having several teeth extracted, called upon her neighbor Mrs. B., to accompany her to the office of the dentist and help her to get her courage up. Reaching the office presently, it was found that Mrs. A's was at a very low ebb, and she was persuaded to try the efficacy of "laughing gas."

The dentist "had given it to scores of patients; there was not the slightest danger," and he assured Mrs. A. that she would recover from the effects of the gas in a little while and would suffer no pain whatever. With nerves wrought up to the highest tension Mrs. A. took the chair, and the dentist began to administer the gas, the effect of which was somewhat startling to him and absolutely horrifying to Mrs. B.

The patient was getting well "under the influence" when the following dialogue occurred:

Mrs. A. Is everything ready?

Mrs. B. Yes, every thing is all right.

Mrs. A. Has the doctor come?

Mrs. B. Yes, the doctor is here.

[Here the doctor gets his nippers on a decayed molar, and after a few twists and jerks, lifts it out.]

Mrs. A. Oh, my! nobody ever suffered such pains; doctor, will it kill me?

Doctor—Oh, no, madam. It will soon be over (as he drops another tooth upon the floor.)

Mrs. A. Where is papa?

At this point Mrs. B's vail was drawn fourteen double over her face, and the dentist's face turned as red as a beet, as he drops out the last ugly tooth and hastily sprinkles a little water in the the lady's face.

In a greatly relieved voice Mrs. A., still laboring under the delusion asks: "Is it a boy or a girl?"—*Courier Journal.*

